# Next-gen sequencing of multi-drug resistant *Acinetobacter baumanii* at Nashville General Hospital at Meharry

**DOI:** 10.1186/1471-2105-12-S7-A14

**Published:** 2011-08-05

**Authors:** Leon Dent, Nahed Ismail, Steven Robinson, Gary Rogers, Siddharth Pratap, Dana Marshall

**Affiliations:** 1 Surgery, Meharry Medical College, Nashville, TN, 37208, USA; 2 Pathology, Meharry Medical College, Nashville, TN, 37208, USA; 3 Student, Hampton University, Hampton, VA, 23668, USA; 4 Electrical Engineering and Computer Science, University of Tennessee, Knoxville, TN, 37996, USA; 5 Microarray & Bioinformatics Core, Microbiology & Immunology, Meharry Medical College, Nashville, TN, 37208, USA

## Background

*Acinetobacter baumannii* is a nonfermentative Gram-negative bacillus, which easily acquires antibiotic resistance determinants and causes life-threatening nosocomial infections [1]. Multi-drug resistant (MDR) strains are common therefore, empirical treatment choices are limited. More knowledge is needed regarding genetic diversity patterns and resistance phenotypes in a given clinical setting. Our goal is to identify the resistance genotypes of *A. baumanii* at Nashville General Hospital and correlate them with MDR phenotypes [1].

## Materials and methods

*A*. *baumannii* isolate MMC#4 is sensitive to tobramycin with a possible extended-spectrum beta-lactamase phenotype. It was compared to *baumannii* reference strains using a next-gen sequencing methodology. Single-end sequencing was conducted on an Illumina Genome Analyzer II system at the Vanderbilt University Genome Technology Core (https://gtc.vanderbilt.edu/gtc/tech). Assembly was conducted at the Meharry Microarray and Bioinformatics Core using BowTie Aligner Software. Gene level annotation was conducted using CuffLINKS software at the University Of Tennessee at Knoxville.

## Results

Initial sequencing yielded 5,250,420 single end reads at 43bp each, totaling 225.76 Mb (Mega bases). The reads were aligned to six MDR *baumannii* reference strains and a fully drug susceptible strain (SDF). Of the 5.2 million total reads, 4.4 million (~85%) aligned to MDR *baumannii* strain ACICU with an average coverage depth of 43.96X fold. Gene level annotation using *A. baumannii* MDR strain AB0057 as a genomic reference revealed sequence reads mapping to 3,209 genes or hypothetical Open Reading Frames (ORFs) of the ~3,800 total genes/ORFs in *baumannii* strain AB0057.

## Conclusions

Strain-to-reference next-gen DNA sequencing of an MDR *baumanii* isolate showed roughly 58% coverage of the ACICU genome by at least one sequence read and a depth of ~44X. Given that the genome size of *A. baumannii* ranges from 3.2Mb in strain STY (sensitive) to 3.9Mb in the MDR AYE strain, we are confident in the proper assembly of a significant portion of the genome. There are six complete assemblies of *A. baumannii* in the NCBI Genome Project data base, as well as ten “in progress”, allowing a true strain-to-reference approach utilizing the already assembled genomes as a scaffold for newly acquired sequences. Although 100% assembly is not likely given the limitations of the short-read sequencing methodology, we would expect to have the majority of the isolate genome unambiguously mapped to a reference strain or assembled into contigs large enough to contribute to the genome databases. The information gained using this technology will lead to rapid and better diagnostics, guide empiric treatment and help people infected with this emerging pathogen.
